# Caveolin 1 is Associated with Upregulated Claudin 2 in Necrotizing Enterocolitis

**DOI:** 10.1038/s41598-019-41442-4

**Published:** 2019-03-21

**Authors:** Guillermo Ares, Christie Buonpane, John Sincavage, Carrie Yuan, Douglas R. Wood, Catherine J. Hunter

**Affiliations:** 1 University of Illinois at Chicago, Department of Surgery, 840S Wood Street, Suite 376-CSN, Chicago, IL 60612 USA; 2 Feinberg School of Medicine, Northwestern University, Department of Pediatrics, 310 East Superior - Morton 4-685, Chicago, IL 60611 USA; 3 Ann and Robert H. Lurie Children’s Hospital of Chicago, 225 E Chicago Avenue, Box 63, Chicago, IL 60611 USA

## Abstract

Necrotizing enterocolitis (NEC) is a devastating gastrointestinal emergency of neonates. Epithelial tight junction (TJ) proteins, such as claudins, are essential for regulation and function of the intestinal barrier. Rho kinase (ROCK) affects cellular permeability and TJ regulation. We hypothesized that TJ protein changes would correlate with increased permeability in experimental NEC, and ROCK inhibitors would be protective against NEC by regulation of key claudin proteins. We tested this hypothesis using an *in vivo* rat pup model, an *in vitro* model of experimental NEC, and human intestinal samples from patients with and without NEC. Experimental NEC was induced in rats via hypoxia and bacteria-containing formula, and in Caco-2 cells by media inoculated with LPS. The expression of claudins was measured by gene and protein analysis. Experimental NEC in rat pups and Caco-2 cells had increased permeability compared to controls. Gene and protein expression of claudin 2 was increased in experimental NEC. Sub-cellular fractionation localized increased claudin 2 protein to the cytoskeleton. ROCK inhibition was associated with normalization of these alterations and decreased severity of experimental NEC. Co-immunoprecipitation of caveolin-1 with claudin 2 suggests that caveolin-1 may act as a shuttle for the internalization of claudin 2 seen in experimental NEC. In conclusion, NEC is associated with intestinal permeability and increased expression of claudin 2, increased binding of caveolin-1 and claudin 2, and increased trafficking of claudin 2 to the cytoskeleton.

## Introduction

Necrotizing enterocolitis (NEC) is the most common intestinal emergency of the newborn population and the leading cause of gastrointestinal morbidity and mortality in preterm infants. The incidence of NEC remains as high as 5–10% of neonatal intensive care unit admissions, with significantly high mortality rates in severe cases^1,2^. Despite decades of research, many details regarding its pathophysiology remain undefined.

It is well documented that infants with NEC have increased intestinal permeability, resulting in increased bacterial translocation, systemic bacteremia, sepsis, and ultimately death^3,4^. The mechanisms leading to increased permeability are poorly understood and are the focus of this study. Gram negative bacteria, such as *Cronobackter sakazakii* (CS), have been implicated in NEC outbreaks in multiple neonatal intensive care units^5^. This and other gram-negative bacteria expressing lipopolysaccharide (LPS) on their cell wall are known to alter intestinal barrier integrity^6^. LPS induces toll-like receptor 4 mediated changes in tight junction (TJ) proteins, leading to increased intestinal permeability^7^. As such, understanding TJ expression and function are an essential area of research in the study of NEC.

TJ are paracellular protein complexes that maintain epithelial barrier structure and function. These transmembrane proteins form intercellular junctions at the lateral border of epithelial cells, creating a permeability limiting seal^8^. TJ proteins have a dynamic function establishing separation between compartments while also selectively regulating paracellular transport. Recent studies show that they are crucial in signal transduction and are modulated by intracellular signaling pathways^9^. There are multiple types of TJ proteins including claudins, occludin, junctional adhesion molecules, and zonula occludens (ZO). Importantly, these proteins differ greatly in function. Some TJ proteins are essential for maintaining intercellular adhesion, such as junctional adhesion molecules (JAM), while others control apicobasal polarization, such as ZO1 and partitioning defective 3 (PAR3)^10^. Claudin proteins are regulators of paracellular ionic conductance via phosphorylation by lysine-deficient protein kinase 1 (WNK 1) and WNK 4. Claudin 2 is a pore-forming TJ, selectively increasing permeability to molecules of certain size or charge, whereas claudin 4 contributes to further strengthening the barrier^11^. In addition to their structural functions, TJ are regulators of multiple intracellular signaling pathways, such as protein kinase C, NF kappa B, and the Rho and Rho kinase (ROCK) pathway. Over-activation of this pathway has been shown to alter expression and localization of TJ proteins and weaken its barrier function^12^. Although the ROCK pathway has been implicated in TJ function, the relationship between ROCK inhibition and claudin TJ proteins has not been well studied. We have previously identified a role of ROCK in protecting against experimental NEC, and in this manuscript we seek to build upon these findings^13^. Herein, we examine the expression of TJ proteins, TJ morphology and function associated with caveolin-1 mediated endocytosis^14,15^, and explore a novel mechanism for ROCK-mediated claudin trafficking. Caveolin-1 is a widely expressed small integral membrane protein that functions in membrane traffic and signal transduction^16^ and has been shown to be involved in the regulation of TJ protein assembly^15,17^, however a specific understanding of its role in NEC has not been described. We focused on the claudin family of TJ proteins given their role as permeability regulators. We hypothesize that pore-forming claudins are overexpressed in NEC, while strengthening ones proteins are under-expressed. Additionally, we hypothesize that ROCK inhibition mitigates the effects of experimental NEC on the alteration of TJ protein expression.

## Materials and Methods

### Bacterial strains and reagents

*Cronobacter sakazakii*, strain BAA-894 (ATCC, Manassas, VA), was grown in Luria broth at 37 °C^18^. Bacteria were centrifuged at 900 g and washed x 2 (VWR, Radnor, PA, USA) before being added to rat pup formula. Lipopolysaccharide (LPS) from *E*. *coli*, strain 0111:B4 (Sigma, St. Louis, MO) was kept as a stock concentration of 5 mg/mL. Rho kinase inhibitor Y-27632 (RI) (Sigma, St. Louis, MO, #Y0503) was resuspended in HyClone water (Fisher, Pittsburgh, PA) to a stock concentration of 43.7 mM.

### Cell culture

The human intestinal epithelial cell line, Caco-2 was obtained from (ATCC, Manassas, VA), passages 18–28. Caco-2 cells were grown in DMEM/F12 media (Gibco, Grand Island, NY) supplemented with 10% fetal bovine serum (FBS) (Gemini, West Sacramento, CA). Cells were seeded on 24-well 6.5 mm transwells (0.4um polycarbonate membane) (Corning, Corning, NY) at 3 × 10^4^ cells per well and on 6-well 24 mm transwells at 7 × 10^5^ cells per well, and grown at 37 °C in a 5% CO_2_ incubator until confluent (7–10 days). Four conditions were studied: control group untreated; NEC group treated with media supplemented with 100 mcg/mL LPS in the basal well; RI group treated with media supplemented with RI at 5uM in the apical and basal wells; and RI + NEC group treated with media supplemented with RI for a 1 hour pre-treatment prior to adding LPS. Concentrations of LPS and Rho kinase inhibitor were chosen on the basis of prior studies and on previously published dose curves^13,19^. The media and reagents were changed every 2 days and cells were collected at various time intervals. Our standard culture media contained 10,000 U/ml of penicillin-streptomycin (Fisher, Pittsburgh, PA), however antibiotics were excluded from the culture media during the experiments. Cells were tested for viability using ApopTag Red *in Situ* Apoptosis Detection Kit (Chemicon, Billerica, MA).

### Cell transfection

Caco-2 cells were seeded in 24-well plates at 10^5^ cells/well. Lipofectamine 2000 (LifeTech, Elmhurst, IL) was used to transfect with the plasmid: Claudin 2 (CLDN2) (NM_020384) Human Tagged ORF Clone, transcript variant 1 (Origene, Rockville, MD, # RG204199) for 72 hours followed by stable selection with gentamycin. Upregulation of claudin 2 was confirmed in western blot with mouse anti-tGFP antibody (Origene, Rockville, MD, # TA150041), and in qRT-PCR with primer: claudin 2 (human) (Santa Cruz, Dallas, TX, # sc-62124-PR). After confirmation of knock-in, cells were cultured with complete media and 5ug/mL gentamycin and treated with LPS for 24 hours versus controls without LPS as per our protocol.

### Rat pup model

Timed-pregnant Sprague Dawley rats were purchased from Charles River Laboratories (Roanoke, IL) and induced near-term at E21 with subcutaneous injection of pitocin 0.1–0.3 units. Rat pups were separated from the dams prior to breast feeding and placed in experimental groups. All pups were exposed twice per day to hypoxia in a 5% O_2_, 95% N_2_ modular chamber (Billups-Rothenberg, Del Mar, CA). Pups were gavage fed three times per day with 0.2–0.3 mL of formula (15 g Similac 60/40 (Ross Pediatrics, Columbus, OH) in 75 mL of Esbilac canine milk replacer (Peg-AG INC., Hampshire, IL). The formula was supplemented for the treatment groups as follows: Clean group fed formula without additional supplementation; NEC group fed formula inoculated with CS bacteria at 1 × 10^7^ concentration once daily; RI group with one of the daily feeds supplemented with 3 mg/kg of RI; and a RI + NEC group fed formula with RI and CS supplementations each day. Rat pups were euthanized on day of life 4 or if they displayed signs of severe NEC in extremis (abdominal distension, discoloration, respiratory distress). Blood and intestines were collected for further analysis. NEC was graded histologically by two independent, blinded reviewers by degree of submucosal edema, neutrophil infiltration, epithelial obliteration, necrosis, and perforation from grade 0 (normal) to grade 3 (severe)^20^. Injury severity score greater or equal to 1.5 was considered NEC. Animals were housed in the Northwestern University facilities that were fully accredited by the Association for Assessment and Accreditation of Laboratory Animal Care International. All procedures were approved by Northwestern University Institutional Animal Care and Use Committee and conducted in accordance with guidelines set forth by the Guide for the Care and Use of Laboratory Animals (IACUC protocol: IS00001655).

### Human tissue

After obtaining approval from the institutional review board (IRB #2013–15152) and informed consent from a parent and/or legal guardian, human intestinal tissue samples were collected from infants undergoing bowel resection at Ann and Robert H. Lurie Children’s Hospital of Chicago, Chicago, IL. All human intestinal tissue samples were collected according to institutional guidelines and regulations. Tissue collected was classified as NEC or control. NEC samples were obtained from neonates undergoing resection for Bell’s stage 3 NEC with perforation or sepsis. Control samples were obtained from patients undergoing bowel resection without active NEC, e.g. ileostomy closure, intestinal atresia, or bowel stricture. Tissue obtained was placed in 10% buffered formalin (Cardinal Health, Dublin, OH), snap frozen in liquid nitrogen, or preserved on optimal cutting temperature (OCT) (Sakura Finetek, Torrance, CA) media at −80 °C. Additional information collected included gestational age, corrected post-conceptual age at time of surgery, gender, indication for operation, and type of tissue obtained.

### Permeability assays

Trans-epithelial electrical resistance (TEER) was measured in Caco-2 cells that were cultured to confluence in 24-well transwell plates. An EVOM2 voltohmeter (World Precision Instruments, Sarasota, FL) was used to measure resistance across the cell layer. After reaching a baseline resistance of 250 Ohm/cm^2^, cells treated according to their experimental group. TEER was measured at serial intervals for all groups and compared to controls for each time point.

100uL of 1 mg/mL 3 kDa fluorescein isothiocynate (FITC) labelled dextran was placed in the apical well and incubated for 2 hours. The basal well media was aspirated and analyzed using a fluorescent plate reader (Molecular Devices Gemini XS, Sunnyvale, CA) to assess FITC concentration. Results were compared with untreated controls at each time point.

Rat pups on day of life 4 were gavage fed 40 mg per 100 g body weight of 10 kDa FITC-dextran. 2 hours later, the rat pups were sacrificed and blood was collected for analysis. Serum concentration of FITC-dextran was assayed with a fluorescent reader plate and compared to the initial concentration given to assess intestinal translocation of FITC-dextran as a marker of permeability.

### Gene expression

Real time quantitative polymerase chain reaction (qRT-PCR) identified gene expression of TJ proteins in Caco-2 cells, rat pup intestines, and human bowel samples. Total RNA was extracted using RNeasy Mini Kit (Qiagen, Valencia, CA). The quality of the RNA was confirmed both by analysis curve on NanoDrop ND-1000 Spectrophotometer (NanoDrop Technologies Inc., Wilmington, DE) and by running an RNA gel to check the integrity of RNA following extraction. 1 ug of RNA was converted to cDNA per reaction using GeneAmp RNA PCR Core Kit (Life Technologies, Carlsbad, CA). In order to confirm there was no contamination of genomic DNA in our prepared cDNA sample, we performed a RT-qPCR with quality check with standard house-keeping genes. Melting curve data revealed that only one product was produced by the primers. Consequently, PCR products were also ran on a DNA gel to confirm the presence of only 1 band per product. qRT-PCR was performed with cDNA using Bio-Rad CFX (Bio-Rad, Hercules, CA) with iQ SYBR Green Supermix (Bio-Rad, Hercules, CA). Cycle threshold gene was normalized with respect to GAPDH gene. Primer sequences are: GAPDH (human) - Forward: 5′-ACCACAGTCCATGCCATCAC-3′, Reverse: 5′-TCCACCACCCTGTTGCTGTA-3′, claudin 1 (human) – Forward: 5′-TGAGGATGGCTGTCATTGGG-3′, Reverse: 5′-AAAGTAGGGCACCTCCCAGA-3′, claudin 2 (human) (Santa Cruz, Dallas, TX, # sc-62124-PR), claudin 3 (human) – Forward: 5′- GCCACCAAGGTCGTCTACTC-3′, Reverse: 5′-CCTGCGTCTGTCCCTTAGAC-3′, claudin 4 (human) – Forward: 5′-GGCCGGCCTTATGGTGATAG-3′, Reverse: 5′-AGTAAGGCTTGTCTGTGCGG-3′, GAPDH (rat) Forward: 5′-ATCACCATCTTCCAGGAGCG-3′, Reverse: 5′-TTCTGAGTGGCAGTGAGGGC-3′, claudin 1 (rat) – Forward: 5′- GCAATGGCGGAAAGTGACAG-3′, Reverse: 5′-ACAAATTCCCACCTCAGCCC-3′, claudin 2 (rat) – Forward: 5′-CGAGAAAGAACAGCTCCGTTT-3′,Reverse: 5′- TTCGCTTGTCTTTTGGCTGC-3′, claudin 3 (rat) – Forward: 5′-GGAAGGCCTGTGGATGAACT-3′, Reverse: 5′-CCTTACGGTCATAGGCGGTG-3′, claudin 4 (rat) – Forward: 5′-CAACTGCATGGAGGACGAGA-3′, Reverse: 5′-TACACATAGTTGCTGGCGGG-3′.

### Protein isolation

Caco-2 cells were washed with phosphate-buffered saline (PBS) (Sigma, St. Louis, MO) and collected by scraping transwells plates. Cells were centrifuged at 2,400 g for 10 minutes at 4 °C. The pellet was re-suspended in cell lysis buffer (Cell Signaling Tech, Boston MA, #9803) and passed through a 27 G needle. After 30 minutes on a rotator at 4 °C, samples were centrifuged at 9,600 g for 15 minutes at 4 °C. The supernanant protein was collected and frozen at −80 °C. Snap frozen rat pup intestine and human tissue samples were sectioned and suspended in cell lysis buffer with 0.25% deoxycholic acid (Sigma, St. Louis, MO). Samples were vortexed for 1 minute and homogenized for 2 minutes in a ground glass tissue grinder on ice. Lysate was centrifuged at 9,600 g for 10 minutes at 4 °C. 6X SDS protein loading buffer (Morganville Scientific, Morganville, NJ) and beta-mercaptoethanol were added to the supernatant, boiled for 4 minutes, and then stored at −80 °C or used for protein analysis.

### Subcellular fractionation

Protein from the four subcellular compartments (cytosol, membrane, nucleus, and cytoskeleton) were extracted from Caco-2 cells and rat pup tissue using ProteoExtract Subcellular Proteome Extraction Kit (EMD Millipore Corporation, Kankakee, IL). Protein concentration in each compartment was assayed using the Bio-Rad Protein Assay kit II (Hercules, CA). We confirmed each subcellular fraction in by western blot analysis with location-specific protein markers: Cytosol – GAPDH (Cell Signaling, Danvers, MA), Membrane – Na-K-ATPase (Cell Signaling, Danvers, MA), Nucleus – Histone H3 (Cell Signaling, Danvers, MA), and Cytoskeleton – Actin (Sigma, St. Louis, MO).

### Antibodies

The following antibodies were used for protein analysis by western blot and immunofluorescence: mouse anti-β-actin (1:50,000^21,22^) (Sigma, Saint Louis, MO, #A5441), mouse anti-claudin 1 (Invitrogen, Carlsbad, CA, #37-4900), mouse anti-claudin 2 (Invitrogen, Carlsbad, CA, #32-5600), rabbit anti-claudin 3 (Invitrogen, Carlsbad, CA, #341700), mouse anti-claudin 4 (Invitrogen, Carlsbad, CA, #329400). The following secondary antibodies were used for detection by immunofluorescence microscopy: Alexa Fluor 594 goat anti-mouse (Invitrogen, Carlsbad, CA, #A-11032) and Alexa Fluor 488 goat anti-rabbit secondary (Invitrogen, Carlsbad, CA, #A-11034).

### Protein analysis

Western blot analysis was used to assay protein expression levels. Isolated protein samples were loaded at equal protein concentration in an SDS-PAGE 12% gel and electrophoresed on a Bio-Rad Mini-PROTEAN 3 apparatus (Bio-Rad, Hercules, CA). Samples were transferred to a nitrocellulose membrane and blocked with 5% non-fat dry milk in PBS with 0.05% Tween for 1 hour at room temperature. The membranes were incubated overnight at 4 °C with primary antibodies diluted to 1:500 in PBS-Tween with 5% milk. After washing with PBS-Tween, secondary antibodies were added for 1 hour followed by Western Blotting Detection Reagent (Amersham, Arlighton Heights, IL). Immunoblot band densities were measured using ChemiDoc XRS + System (Bio-Rad, Hercules, CA) and analyzed with Image Lab Software (Bio-Rad, Hercules, CA).

### Immunofluorescence microscopy

Caco-2 cells grown on chamber slides were stained for apoptosis using the ApopTag Red *In Situ* Apoptosis Detection Kit (Chemicon, Billerica, MA). Caco-2 cells grown on transwell membranes and cryosections of rat and human tissue were washed with PBS and blocked with 10% normal goat serum in PBS with 0.1% triton. The slides were then incubated in primary antibody diluted in PBS to 1:300 at 4 °C overnight. After washing with PBS, slides were incubated with respective secondary antibodies diluted to 1:2000 for 1 hour. DAPI conjugated fluoroshield (Sigma, St. Louis, MO) was applied to each slide and mounted with a glass cover slip. Multiphoton microscopy was performed on a Nikon A1R multiphoton microscope and Nikon Eclipse 90i. All images were analyzed with Fiji ImageJ 1.51n^23^

### Statistical analysis

All experiments described above were performed at least in triplicate. Graphs were generated using Microsoft Excel or GraphPad Prism 7 (La Jolla, CA). Statistical significance of the data was analyzed using Student’s *t* test or ANOVA where appropriate between groups and difference was considered significant at p < 0.05. Outliers greater than two standard deviations were removed. The datasets generated during and/or analyzed during the current study are available at the discretion of the corresponding author upon reasonable request.

## Results

### Permeability is increased in experimental NEC

**Figure 1:**
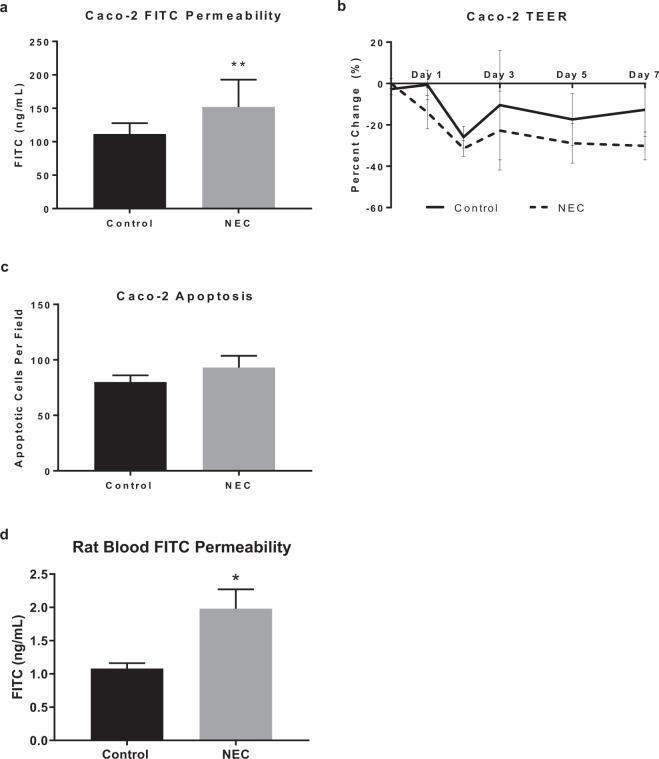
Increased permeability in experimental NEC. (**a**) Caco-2 cells treated with LPS for 7 days (NEC) have increased permeability to 3 kDa FITC-dextran (n = 15). (**b)** LPS treatment in Caco-2 cells decreased TEER progressively from day 1 (−1% control vs. −14% NEC, ***p < 0.001) through day 7 (−13% vs. −30%, **p < 0.01) (n = 23). (**c)** Caco-2 cell immunofluorescent DNA fragmentation staining apoptotic cell count per 1000 cells showed no difference between controls and LPS-treated cells (p = 0.32, n = 10). (**d)** The combination of hypoxia and formula feedings without (control) or with *Cronobacter sakasakii* bacteria (NEC) results in increased translocation of gavage fed FITC-dextran through the intestinal epithelium into the blood. Serum from rat pups was collected and permeability was assessed by FITC-assay (n = 9 control, 6 NEC). Outliers greater than 2 standard deviations were excluded. All values are mean ± SEM (*p < 0.05, **p < 0.01, ***p < 0.001).

To determine the effect of LPS on the permeability of monolayers of human intestinal epithelial cells (Caco-2) we assayed the transit of FITC-dextran in response to LPS treatment. Exposure of Caco-2 cells resulted in an increased permeability FITC-dextran compared to controls at 24 hours (p < 0.005) (Fig. 1a). In addition to FITC-dextran assay, TEER was measured at set intervals during a 7 day period. Cells treated with LPS had a 14% decrease in TEER by 24 hours post exposure to LPS, compared to 1% decrease in controls (p < 0.005). The TEER gap between the two groups widened progressively, peaking at day 7 with experimental NEC cells having a 23% larger decrease in TEER compared to controls (p < 0.005) (Fig. 1b). To ensure that changes in permeability were not a consequence of increased cellular death, apoptosis was detected using DNA fragmentation assay. There was no significant difference in apoptosis seen between controls and LPS-treated Caco-2 cells at the time points studied, seen in Fig. 1c. To determine whether similar alterations in permeability are associated with the *in vivo* rat pup model of NEC, we performed a 4 day time course under control (clean formula) and NEC (hypoxia + CS inoculated formula) conditions. At the conclusion of the experiment, pups were gavage fed FITC-dextran and blood was collected to assess translocation of FITC-dextran through the intestinal barrier and into the systemic circulation. Rats with evidence of bowel perforation were excluded from this analysis. Compared to controls, there was a two-fold increased permeability in experimental NEC rats (p < 0.05), as shown in Fig. 1d. Thus, NEC conditions increased permeability in both our *in vitro* and *in vivo* model.

### Claudin changes in Caco-2 NEC

**Figure 2:**
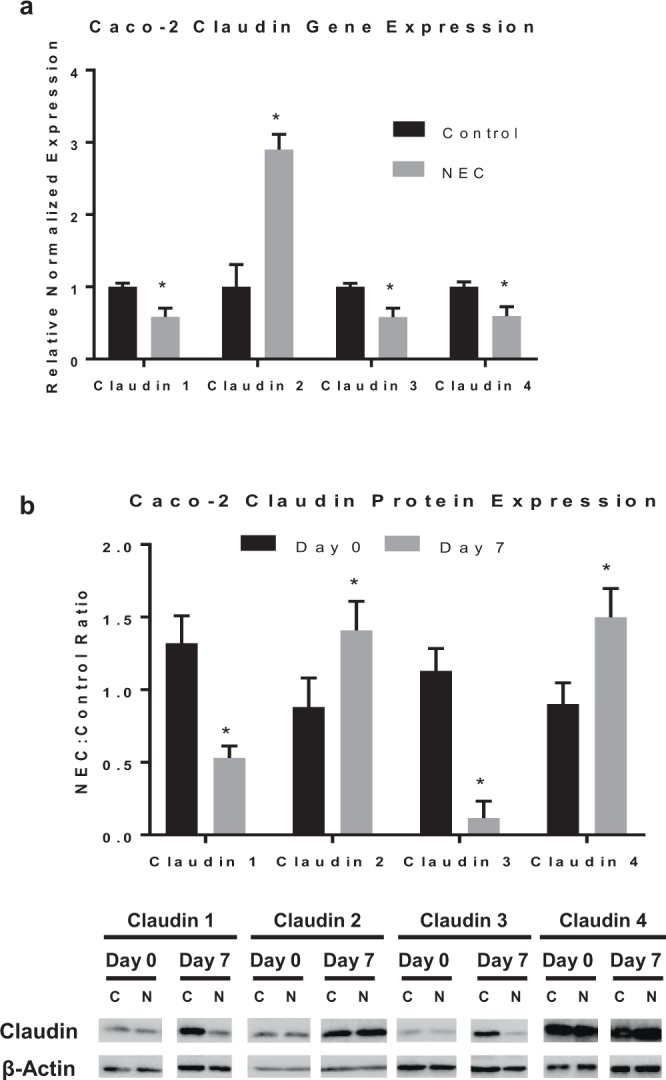
Claudin proteins are altered in response to LPS in Caco-2 cells. Treatment of Caco-2 cells with LPS for 7 days induces changes in claudin gene and protein expression. (**a)** qRT-PCR showed experimental NEC decreased gene expression of claudins 1, 3, and 4 (n = 3/5/5, respectively), and increased claudin 2 expression (n = 9). Genes were normalized to GAPDH. (**b)** Western blot analysis showing ratio of claudin protein expression in experimental NEC cells to same-day controls at days 0 and 7. Protein expression of claudin 1 and 3 is decreased in experimental NEC, while claudin 2 and 4 expression is increased. Below, representative immunoblots (full blots included in Supplementary Fig. S1) probed with antibodies against each claudin on day 0 and 7 in control (C) and LPS-treated cells (N); blots were reprobed with antibodies against β-Actin; analyzed in at least three separate experiments. All values are mean ± SEM (*p < 0.05).

Claudins are essential in the maintenance of intercellular adhesion and barrier function^8^; therefore, we evaluated whether differences in expression were found within the claudin family in our *in vitro* model. Analysis with qRT-PCR yielded several different patterns of claudin expression. Specifically, Caco-2 cells exposed to LPS for 7 days had decreased claudin 1, 3, and 4 expression compared to controls (p < 0.0005 for each). Conversely, an increase in claudin 2 was found in cells exposed to experimental NEC conditions (p < 0.0005) (Fig. 2a). To determine whether observed changes in RNA level correlated with altered translation, we measured claudin protein expression over a 7 day time course. Western blot analysis identified the differences in protein expression for each claudin on each day. Figure 2b demonstrates a significant decrease in claudins 1 and 3 protein in experimental Caco-2 cells by Day 7 compared to the baseline at Day 0 (p < 0.05). In contrast, claudin 2 was increased in experimental NEC cells on Day 7 compared to Day 0 (p < 0.05). These changes in protein expression correlate with the gene expression profile for all claudins except claudin 4, shown in Fig. 2a. Interestingly, the NEC:Control protein ratio was increased on Day 7 compared to Day 0 for claudin 4 protein expression (p < 0.05), while the gene expression was decreased in experimental NEC. These findings suggest that the pore-forming claudin 2 protein is overexpressed in NEC while the barrier-strengthening claudins 1 and 3 may be under-expressed.

### Claudin 2 expression is increased in the model of rat pup NEC

**Figure 3:**
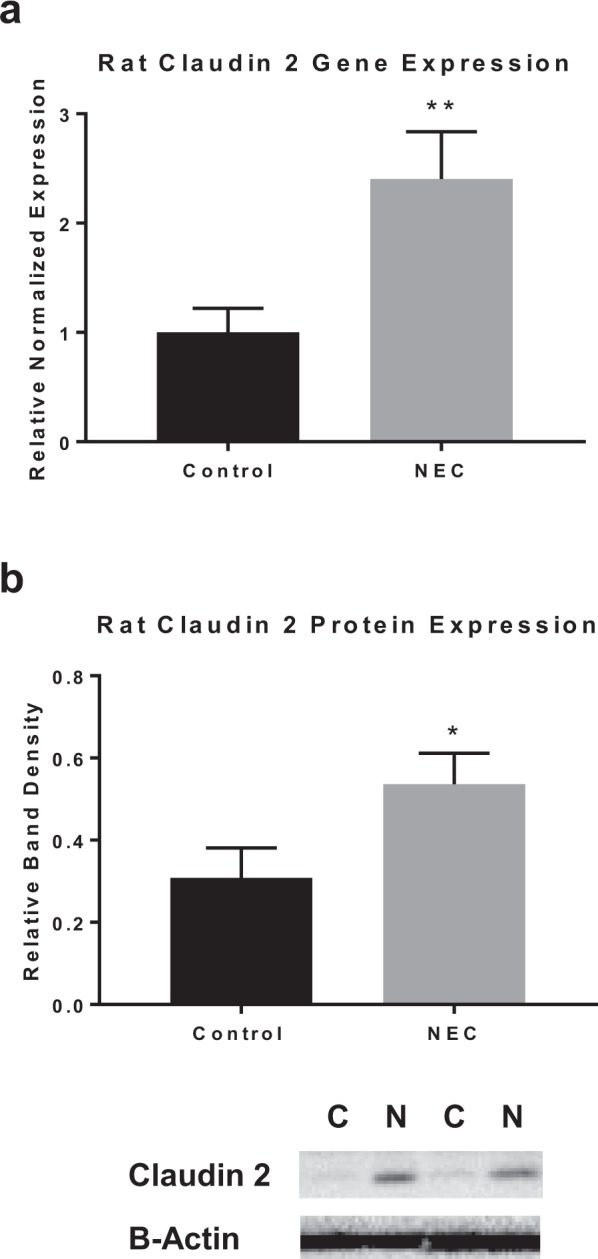
Increased claudin 2 expression in rats with experimental NEC. (**a)** Preterm rat pups gavage fed formula containing CS bacteria to induce experimental NEC compared to controls had increased claudin 2 gene expression by qRT-PCR (n = 8). (**b)** Western blot densitometry graph shows increased claudin 2 protein expression in experimental NEC rats vs. controls (n = 5); representative immunoblots for claudin 2 in bottom panel for control (C) and NEC rats (N); blots were reprobed with β-Actin (full blots included in Supplementary Fig. S2). All values are mean ± SEM (*p < 0.05, **p < 0.01).

To determine whether the alterations identified in claudin 2 in the *in vitro* model correlated with an established *in vivo* model of NEC, we tested the rat pup model of NEC. We postulated that we would identify similar changes in claudin 2 in rats with NEC compared with controls. Claudin 2 gene expression from rat intestines was quantified using qRT-PCR. Similar to our *in vitro* results, rat pup intestines in the experimental NEC group had an overexpression of claudin 2 compared to controls animals of the same age (p < 0.005) (Fig. 3a). Western blot analysis also demonstrated an increase in claudin 2 protein expression in the NEC group compared to control (p < 0.05) (Fig. 3b). Therefore, our findings in the animal and cellular model both show an upregulation of claudin 2 in experimental NEC.

### Defining claudin 2 localization in experimental NEC

**Figure 4:**
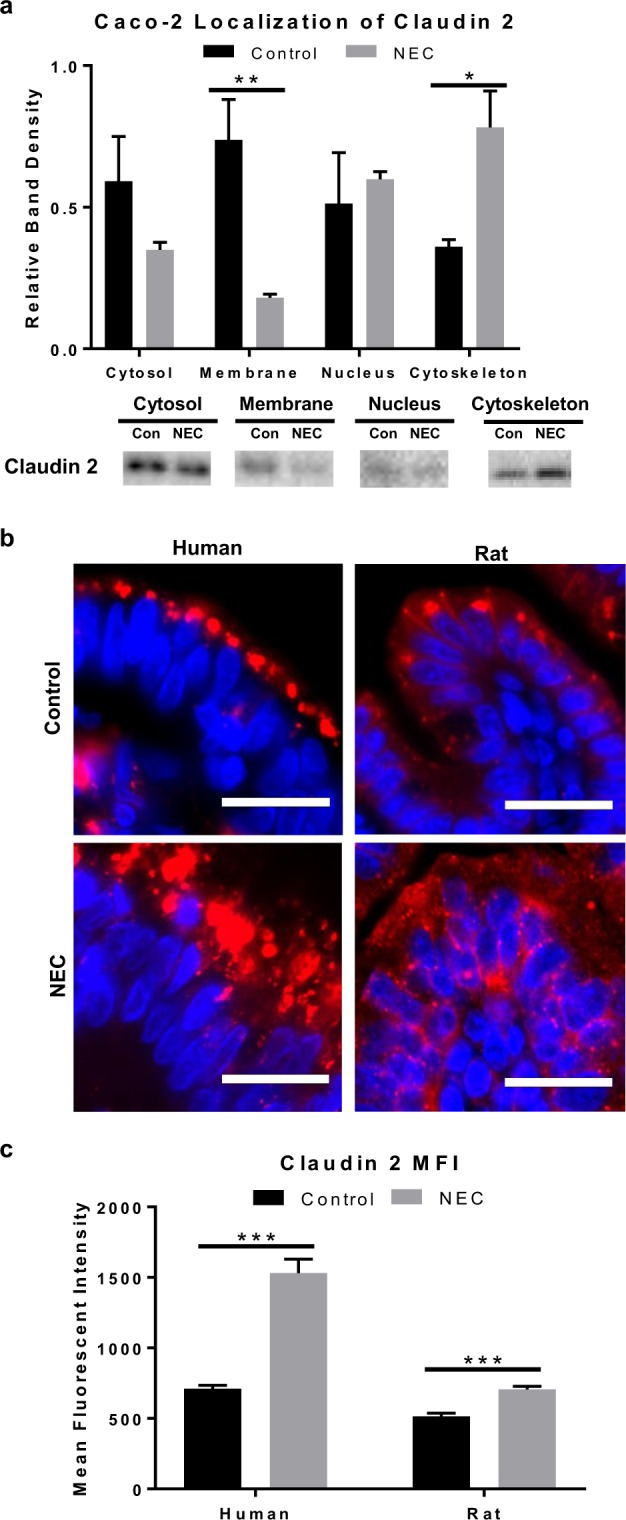
Human and experimental NEC is associated with changes in claudin 2 localization within the cell. (**a)** Caco-2 cells treated with LPS for 7 days were fractionated into subcellular compartments and analyzed with western blot showing a decrease in claudin 2 expression in the membrane of LPS-treated cells vs. controls (n = 5/4) and increased expression in the cytoskeleton vs. controls (n = 3/4); representative immunoblots for claudin 2 per compartment shown (full blots included in Supplementary Fig. S3). (**b)** Representative immunofluorescence micrographs of human and rat intestinal villi stained for claudin 2 (red) show focal expression at the cell membrane in control groups vs. internalized pattern of expression in NEC groups; nuclei stained with DAPI (blue), scale bar = 20 uM. (**c)** Mean fluorescent intensity measured in 15 × 30 um size boxes (2-cells wide) showed increased claudin 2 fluorescence in humans and rats NEC compared to controls (n = 3/3). All values are mean ± SEM (*p < 0.05, **p < 0.01, ***p < 0.001).

Since both the quantity of claudin protein expression and its location within the cell may alter function, we performed subcellular fractionation to determine localization during NEC conditions. Western blot analysis demonstrated a decrease in the expression of membrane bound claudin 2 protein in experimental NEC cells compared to controls (p < 0.05) (Fig. 4a). This was accompanied by increased expression in the cytoskeleton compartment (p < 0.05). There was no difference in claudin 2 expression in the cytosol or the nucleus. To confirm whether similar findings occurred *in vivo* NEC, we performed immunofluorescence of claudin 2 in human and rat pup intestines. Indeed, we identified differences in the pattern of claudin 2 staining between control samples and those with NEC. Specifically, claudin 2 in control human samples and animals showed crisp and localized staining at the cell-cell borders and the membrane surrounding enterocytes (Fig. 4b). However, the NEC groups had markedly increased expression of claudin 2, with a diffuse pattern that was less localized to the cell surface membrane. Mean fluorescent intensity was calculated and showed a significant increase in claudin 2 staining in NEC human samples and NEC + rats (p < 0.0005) (Fig. 4c). NEC is associated with both an increase in expression and internalization of claudin 2 protein compared to controls.

### Claudin 2 overexpression precedes changes in permeability

**Figure 5:**
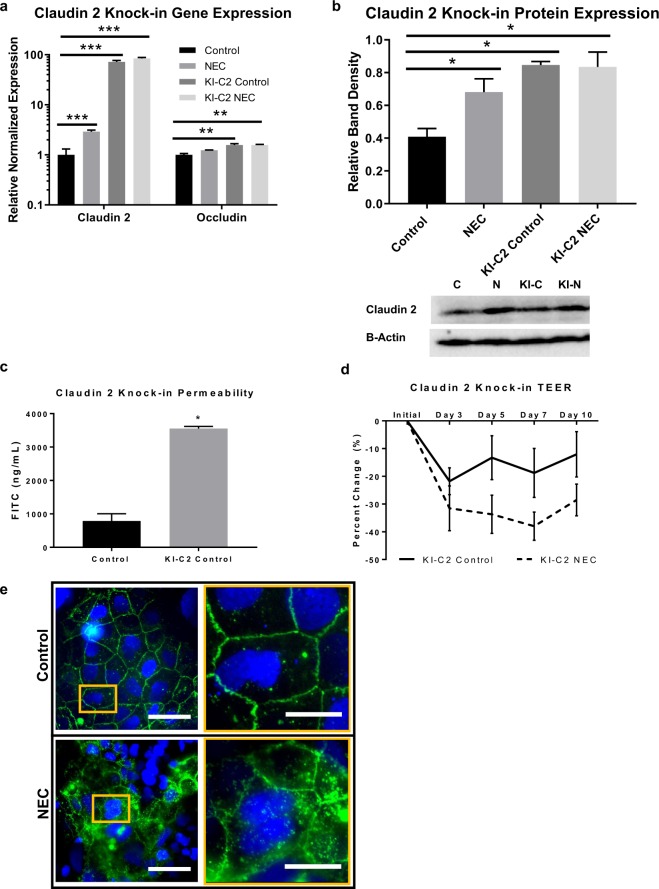
Claudin 2 knock-in confers increased permeability in Caco-2 cells. (**a)** Caco-2 cells with claudin 2 knock-in have >70-fold greater gene expression of claudin 2 on qRT-PCR compared to wild type controls (n = 3 per group); KI-C2, Claudin 2 Knock-in. Occludin gene expression was less than 2-folds increased in knocked-in cells. (**b)** Western blot band densitometry graph confirms increased claudin 2 expression in knock-in cells compared to controls (n = at least 3 per group); representative immunoblots (full blots included in Supplementary Fig. S4) for claudin 2 were then probed with β-Actin (C, Control; N, NEC; KI-C, knock-in control; KI-N, knock-in NEC). (**c)** Permeability to 3 kDa FITC-dextran was increased in claudin 2 knock-in cells compared to controls (n = at least 9 per group). (**d)** TEER assay showed a trend towards further increased permeability in the claudin 2 knock-in cells after exposure to LPS, with the most pronounced effects after 5 days of exposure (p = 0.10). (**e)** Immunofluorescence microscopy of claudin 2 knock-in cells with green fluorescent tag shows focal expression of claudin 2 at the cell membrane that changes to an internalized localization with exposure to experimental NEC; left column scale bar = 20 uM scale bar, magnified inset (right column) scale bar = 50 uM; nuclei stained with DAPI (blue). All values are mean ± SEM (*p < 0.05, **p < 0.01, ***p < 0.001).

To determine whether changes in permeability occur as a result of upregulation of claudin 2, we generated a genetic claudin 2 knock-in using Caco-2 cells. Upregulation of claudin 2 was confirmed by qRT-PCR in Fig. 5a, showing significantly increased gene expression in the knock-in groups compared to the wild-type Caco-2 cells (p < 0.0001). To control for alterations in the expression of other TJ in response to this knock in we measured the gene expression of additional TJ proteins, which revealed only a biologically non-significant upregulation of occludin. The small increase in occluding in the knock-in cells was of markedly inferior magnitude to that of claudin 2. Protein expression of claudin 2 was significantly increased in the knock-in cells compared to controls, Fig. 5b. We tested functional changes associated with the knocked-in claudin 2 and found increased permeability to FITC-dextran compared to controls (p < 0.05), shown in Fig. 5c. Exposure to NEC conditions led to greater increases in permeability over 10 days with a trend towards decreased TEER in knock-in cells exposed to LPS compared to knock-in controls, Fig. 5d. Given the observed changes in the subcellular location of claudin 2, described previously in Fig. 4, we performed immunofluorescence to assay localization in the setting of claudin 2 over expression. Figure 5e shows a similar pattern of distribution in claudin 2 knock-in cells, and exposure to NEC conditions led to an internalized pattern of expression with more claudin 2 localizing to the cytoskeleton and less in the membrane compared to knock-in controls. These changes correlate with results from subcellular protein analysis in Fig. 4a. Taken together, these data suggest that claudin 2 overexpression leads to increased permeability and that exposure to experimental NEC induces internalization of claudin 2.

### Rock inhibition is protective against NEC

**Figure 6:**
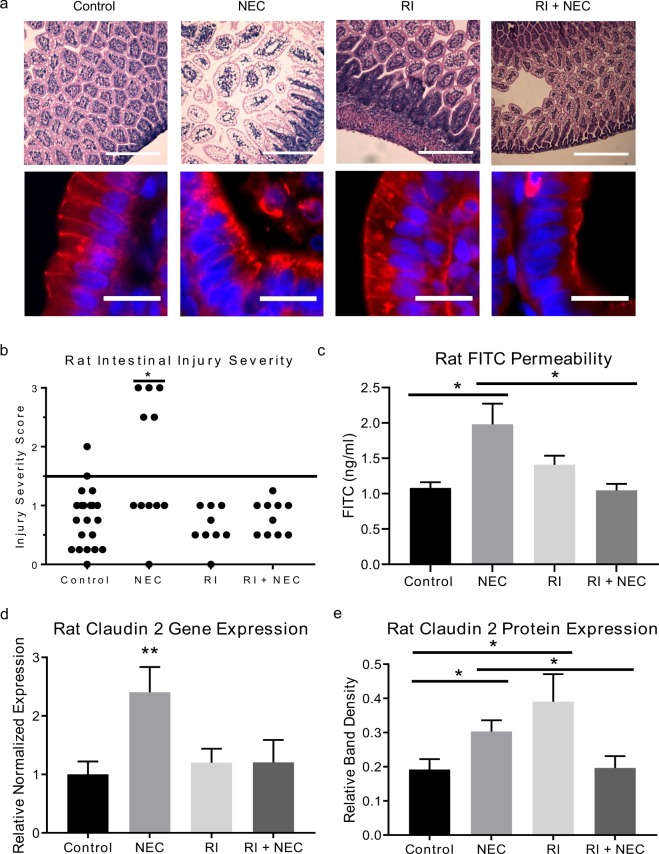
ROCK inhibition is protective against experimental NEC. (**a)** (Top) Hypoxia exposure and gavage feeding of rats with bacteria-inoculated formula led to histological development of NEC compared to controls fed clean formula; pups fed ROCK inhibitor (RI) and those pretreated with RI before exposure to NEC formula (RI + NEC) had no histological changes compared to controls; scale bar = 200 uM. (Bottom) Representative immunofluorescence micrographs of rat intestinal epithelium stained for claudin 2 (red); nuclei stained with DAPI (blue); scale bar = 20 uM. In control rats, claudin 2 expression is focused at the cell membrane. In rats with NEC, claudin 2 demonstrates a spiculated internalized pattern of expression. With RI treatment, the claudin 2 expression is highly expressed, with maintained organization within the cell. RI + NEC treatment shows diminished claudin 2 expression; but a maintained organized pattern. (**b)** Intestinal injury was graded histologically based on severity of mucosal erosion, submucosal edema, and derangement of villi architecture; NEC was defined as score >1.5. (**c)** RI treatment in the rat pup model prevented NEC induced intestinal permeability changes. Compared to NEC rats, RI + NEC rats had significantly lower permeability (p < 0.05). (**d**,**e)** qRT-PCR for claudin 2 demonstrated increased gene expression in NEC vs. controls, while rats pretreated with RI before NEC exposure did not have a change in claudin 2 expression. Western blot densitometry for claudin 2 demonstrated increased expression in NEC and RI treated rats. Rats pretreated with RI before NEC exposure did not have a change in claudin 2 expression as seen in the NEC group. Values are mean ± SEM of at least three separate experiments with three animals per group (*p < 0.05, **p < 0.01).

Rho kinase inhibition has been found to have a role in regulation of other TJ proteins, such as occludin^24^, therefore we hypothesized that ROCK inhibition would have a protective effect against experimental NEC by preventing upregulation of claudin 2. To test this hypothesis rat pups were fed clean formula, formula with CS bacteria to induce NEC, clean formula with RI, or CS formula with RI for four days prior to collection of intestines. Figure 6a shows rat intestines that were sectioned and stained with hematoxylin and eosin to measure disease severity. An injury severity score was assigned by two reviewers blinded to groups. Intestines of rats exposed to RI and RI + NEC conditions maintained normal villus architecture, had minimal epithelial sloughing, and gross intestinal integrity similar to that of control rats. This improved appearance correlated with low injury severity scores. The injury severity scores in experimental rat pups compared with controls was markedly increased (p < 0.05), RI (p < 0.005), and RI + NEC rats (p < 0.05) (Fig. 6b). Furthermore, the subcellular distribution of claudin 2 varies between experimental groups. The intestinal epithelium of rats with NEC demonstrate a spiculated internalized pattern of claudin 2 expression as opposed to the linear expression seen at the cell membrane in control pups (Fig. 6a). In those pups treated with RI, claudin 2 is highly expressed with maintained organization within the cell. RI + NEC treatment shows decreased claudin 2 expression; however, with maintained organization within the epithelium. Furthermore, RI treatment prevented the NEC-associated increased intestinal permeability, compared to rats with NEC, RI + NEC rats had significantly lower permeability (p < 0.05) (Fig. 6c). Measurement of claudin 2 gene expression by qRT-PCR revealed an increased expression in NEC pups compared to controls, but not in RI or RI + NEC (p = 0.002) (Fig. 6d). Analysis with western blot further confirmed an increased expression of claudin 2 protein in experimental NEC rats compared to controls (p < 0.05) and compared to RI + NEC (p < 0.05) (Fig. 6e). Taken together, these results indicate that RI has a protective effect on the intestinal epithelium against experimental NEC and normalizes the claudin 2 expression and localization.

### Claudin 2 association with caveolin-1

**Figure 7:**
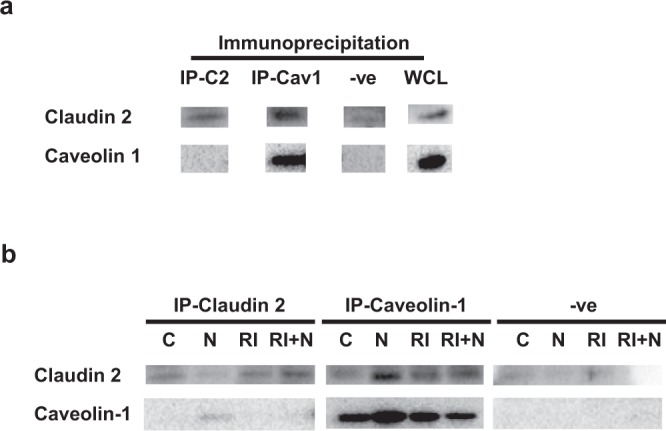
Claudin 2 co-immunoprecipitates with caveolin-1. (**a)** Western blot in control rats showing expression of claudin 2 and caveolin-1 in in the following samples: IP-C2, intestines immunoprecipitated with an anti-claudin 2 antibody; IP-Cav1, immunoprecipitation with an anti-caveolin-1 antibody; −ve, negative controls immunoprecipitated with an irrelevant IgG; WCL, whole cell lysate of rat intestines without immunoprecipitation for positive control. (**b)** Representative immunoblots (full blots included in Supplementary Fig. S5) of intestine samples immunoprecipitated with claudin 2, caveolin-1, and negative control antibody showing expression of claudin 2 and caveolin-1 in rats exposed to control (C), NEC (N), ROCK inhibitor (RI), and ROCK inhibitor plus NEC (RI + N).

Internalization of other TJ proteins such as occludin, has been associated with caveolin shuttling after exposure to bacterial toxins^12,15^. Therefore, we sought to investigate the relationship between claudin 2 and caveolin-1 as a potential mechanism for the internalization of claudin 2 seen in experimental NEC. Claudin 2 expression was detected in control rat intestine lysates that had been immunoprecipitated using anti-caveolin 1 antibody, suggesting an association between caveolin-1 and claudin 2 (Fig. 7a). Immunoprecipitation with a negative control, did not detect claudin 2 or caveolin-1. We observed an increased association of caveolin-1 and claudin 2 in NEC (Fig. 7b). However, we found that the affinity for claudin 2 to bind to caveolin-1 is less than the affinity for caveolin-1 to bind to claudin 2 in NEC. Treatment with RI decreased the association of caveolin-1 with claudin 2. Interestingly, these findings indicate that there is an increase in binding between caveolin-1 and claudin 2 in NEC.

## Discussion

NEC is a severe disease of the gastrointestinal system affecting mostly premature infants. Its pathogenesis is multifactorial, involving activation of the immune system, an aggressive inflammatory response, and degradation of the bowel mucosa^25,26^. Alterations in TJ proteins and epithelial barrier function have been linked to human disease, such as infectious enteritis and inflammatory bowel disease^27^. While we acknowledge that the development and progression of NEC involves a complex interaction between the immune system, inflammation and changes in the structure and function of the intestinal epithelium, we narrowed the focus of our study to protein level changes occurring during this disease state. To better understand the role of specific TJ proteins in NEC, multiple animal and cellular models have been established^28^. CS bacteria, which express LPS in their cell wall, has been demonstrated to disrupt TJ in experimental NEC models using rats^18^ and Caco-2 cells^29^. The addition of LPS to cell cultures creates an inflammatory response akin to that seen in human NEC leading to mucosal injury^19^. We found that these models exhibit increased permeability across the intestinal epithelial layer.

Intercellular permeability is regulated by TJ proteins via intra- and extra-cellular signaling pathways^8^. Claudins are transmembrane proteins that create a seal, binding with the extracellular portion of other TJ proteins. The intracellular domains of TJ proteins interact with cytosolic proteins including actin, creating a link to the cytoskeleton^30^. Other authors have shown that perijunctional actin contraction induced by myosin light chain alters permeability by changing the configuration of TJ proteins^31^. Claudins also have varying functions within the cell, including regulation of cation selectivity, water, movement of inflammatory mediators, and cellular proliferation and self-renewal^32,33^. We have screened the following proteins: claudin 1–4, and zona occludins for their effects in NEC. Significant changes were only reliably identified in claudin 2 and claudin 4 and therefore the focus of our results and discussion will focus on these areas. Claudin 2 has been identified as a pore-forming protein in multiple organs, particularly in those that require significant paracellular transport, such as the intestine, liver, and kidneys^34,35^. Intestinal permeability is essential for the absorption of nutrients. Neonates in particular have high permeability in their gut that decreases as their bowel matures and, presumably, as their nutritional needs change. The profile of claudins in the intestine varies as humans age, with a higher proportion of claudin 2 expressed in the perinatal period than later in infancy^27^. Moreover, increased claudin 2 expression is found in inflammatory disease states and contributes to barrier dysfunction^36^. While claudin 2 is not expressed in normal colonic epithelium, previous authors have found claudin 2 expression in the diseased colons of patients with ulcerative colitis and Crohn’s disease. Moreover, microscopy has demonstrated damaged TJ strands in these disease states with significant upregulation of claudin 2^37^. We found overexpression of claudin 2 gene transcripts and protein in rat pups and cells with experimental NEC. Since our cellular model is devoid of an immune system to mediate or exacerbate the inflammatory response, we believe that the changes in permeability found in our line of experiments are largely associated with epithelial barrier dysfunction. The genetic claudin 2 knock-in supports the conclusion that increased intestinal permeability is a downstream effect from the upregulation of claudin 2 seen as a result of NEC. Not only was claudin 2 overexpressed in our NEC groups, but there was also a change in localization within the subcellular compartment. We found increased claudin 2 expression in the cytoskeleton of the cell and decreased amount in the membrane. We speculate this change in claudin 2 distribution might be the result of increased production in the cell and increased transit to the membrane, as the ongoing inflammatory stimulus induces overproduction of claudin 2. Interestingly, we found increased claudin 4 protein expression, though there was decreased gene expression in NEC. We expected to find decreased claudin 4 expression, given its role as a barrier-strengthening claudin. While this discrepancy with claudin 4 was unexpected based on findings from other studies, it is possible that changes in claudin 4 protein expression happen at a different time point than Day 7. In fact, our time course showed a non-significant trend towards decreased claudin 4 NEC:Control ratio at prior to Day 7.

Various studies have examined the role of protective enteral supplements against NEC. Human breast milk has been shown to prevent degradation of barrier-strengthening TJ proteins in NEC^38,39^. Barrier integrity has also been preserved with ingested probiotics, which resulted in decreased permeability and severity of NEC^40^. Upregulation of claudin 2 was found in an experimental mouse model of NEC, which also showed preserved permeability with the administration of a probiotic^41^. Therefore, the role of ingested pharmaceuticals in the preservation of barrier function was of special interest to us. Rho kinase inhibitors, which have been already used in humans for the treatment of arterial vasospasm^42^, have been found to have regulatory effects on TJs^43^. Other authors have also shown decreased intestinal injury from hypoxia and reoxygenation in rats using RI^44^. Therefore, we gavaged RI and found similar protective effects in the rat gut in our experimental NEC model. Activation of the Rho kinase signaling pathway is associated with disruption of the cytoskeleton and loss of tight junction organization^43^. With inhibition of this pathway, there is reversal of the disaggregation of the cytoskeleton, allowing for improved tight junction organization and enhanced functionality of claudin 2. In a normal physiologic setting (control rats) we found RI treatment to cause an increase in the expression and functionality of claudin 2, likely due to complex interactions with the preserved actin cytoskeleton and other adherens junction proteins^45^. When RI treatment was administered to rats under NEC-inducing conditions, we found RI to be protective against the internalization and disaggregation of claudin 2 in the cell membrane.

Additionally, we found increased binding between caveolin-1 and claudin 2 in NEC. We propose a potential mechanism for these findings: (1) Claudin 2 is upregulated in response to CS/inflammatory antagonists/NEC. (2) The increased amount of claudin 2 allows for increased binding to caveolin-1. (3) Increased caveolin-1 mediated endocytosis of claudin 2 redistributes the TJ protein inside the cell, mainly to the cytoskeleton. (4) Caveolin-1 is responsive to actin cytoskeleton stress fiber formation under the RhoA pathway through filamin A. Therefore, RI prevents disaggregation of the actin cytoskeleton, reducing the amount of claudin 2 trafficking inside the cell. Although the association of caveolin 1 and tight junction proteins has been implicated in intestinal epithelial disease states, the exact mechanism is still not understood. Further research is needed to elucidate the complex interactions between ROCK, caveolin-1, the cytoskeleton and tight junction proteins in NEC.

## Supplementary information

Supplemental Information
