# Supplementary material for: Caveolin 1 is Associated with Upregulated Claudin 2 in Necrotizing Enterocolitis
Source: Sci Rep. 2019 Mar 21;9:4982. doi: 10.1038/s41598-019-41442-4 (PMC6428816; doi:10.1038/s41598-019-41442-4)
Supplement: Supplementary file 1 — Supplemental Information [file 41598_2019_41442_MOESM1_ESM.pdf]

Supplementary Information

Title: Caveolin 1 is Associated with Upregulated Claudin 2 in Necrotizing Enterocolitis

Authors: Guillermo Ares, Christie Buonpane, John Sincavage, Carrie Yuan, Douglas R. Wood, Catherine J. Hunter

Caco-2 Claudin Protein Expression

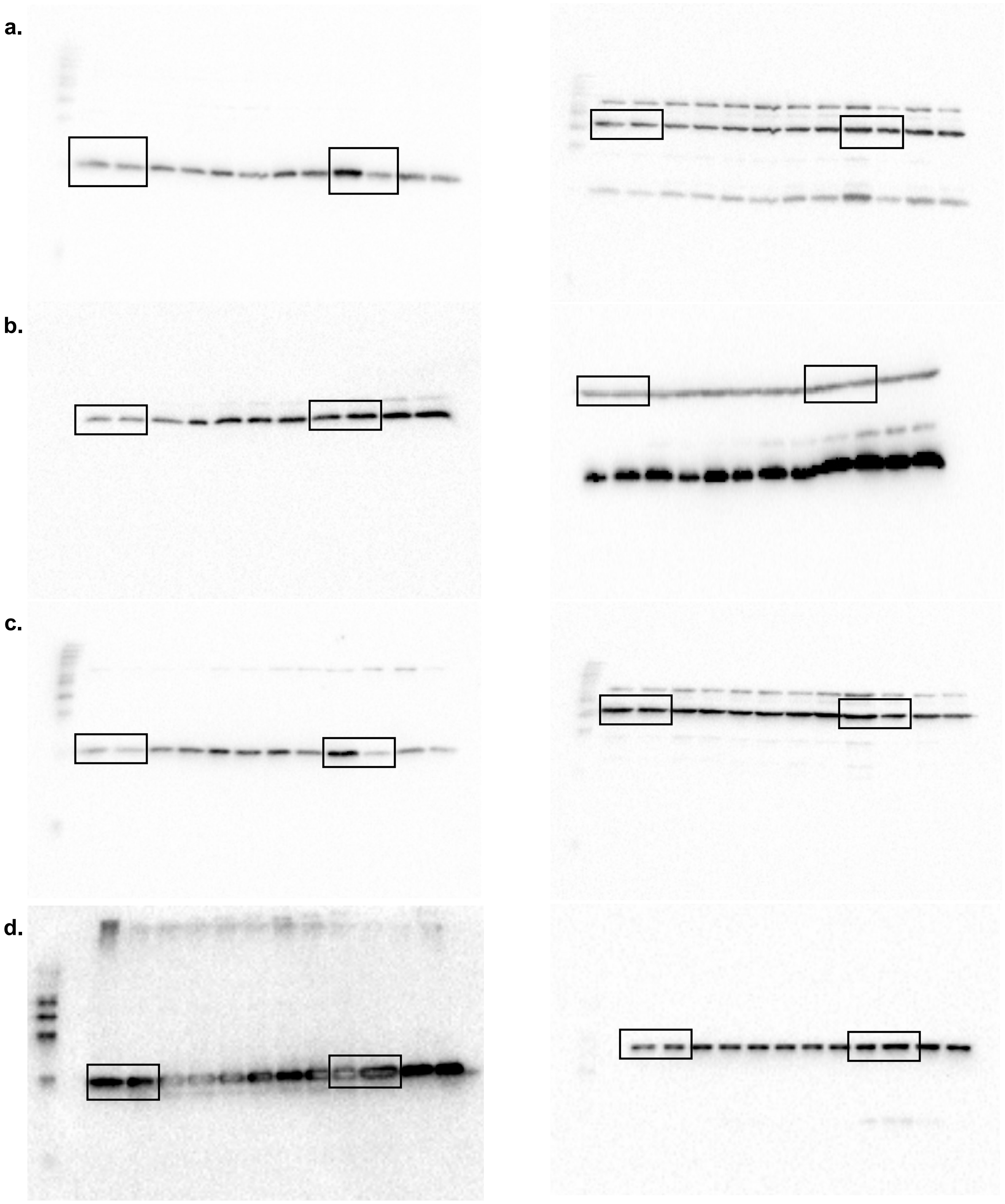

Supplementary Figure S1: Full length western blots used for Figure 2b. **a.** Caco-2 Claudin 1 (left) and β-actin (right) western blots. **b.** Caco-2 Claudin 2 (left) and β-actin (right) western blots. **c.** Caco-2 Claudin 3 (left) and β-actin (right) western blots. **d.** Caco-2 Claudin 4 (left) and β-actin (right) western blots.

Rat Claudin 2 Protein Expression

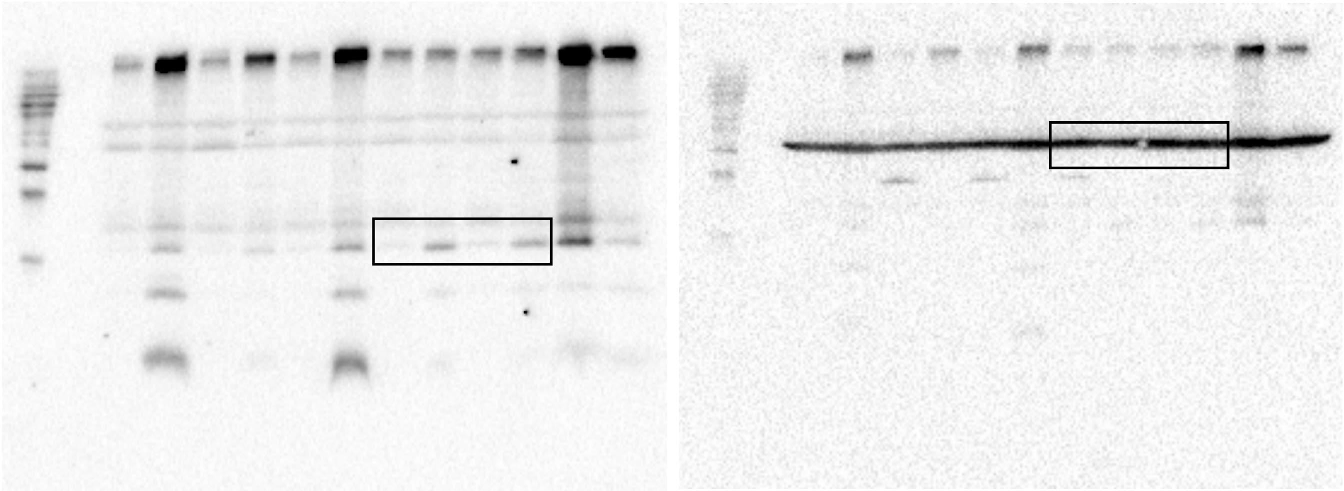

Supplementary Figure S2: Full length western blots used for Figure 3b. Rat Claudin 2 (left) and β-actin (right) western blots.

Caco-2 Localization of Claudin 2

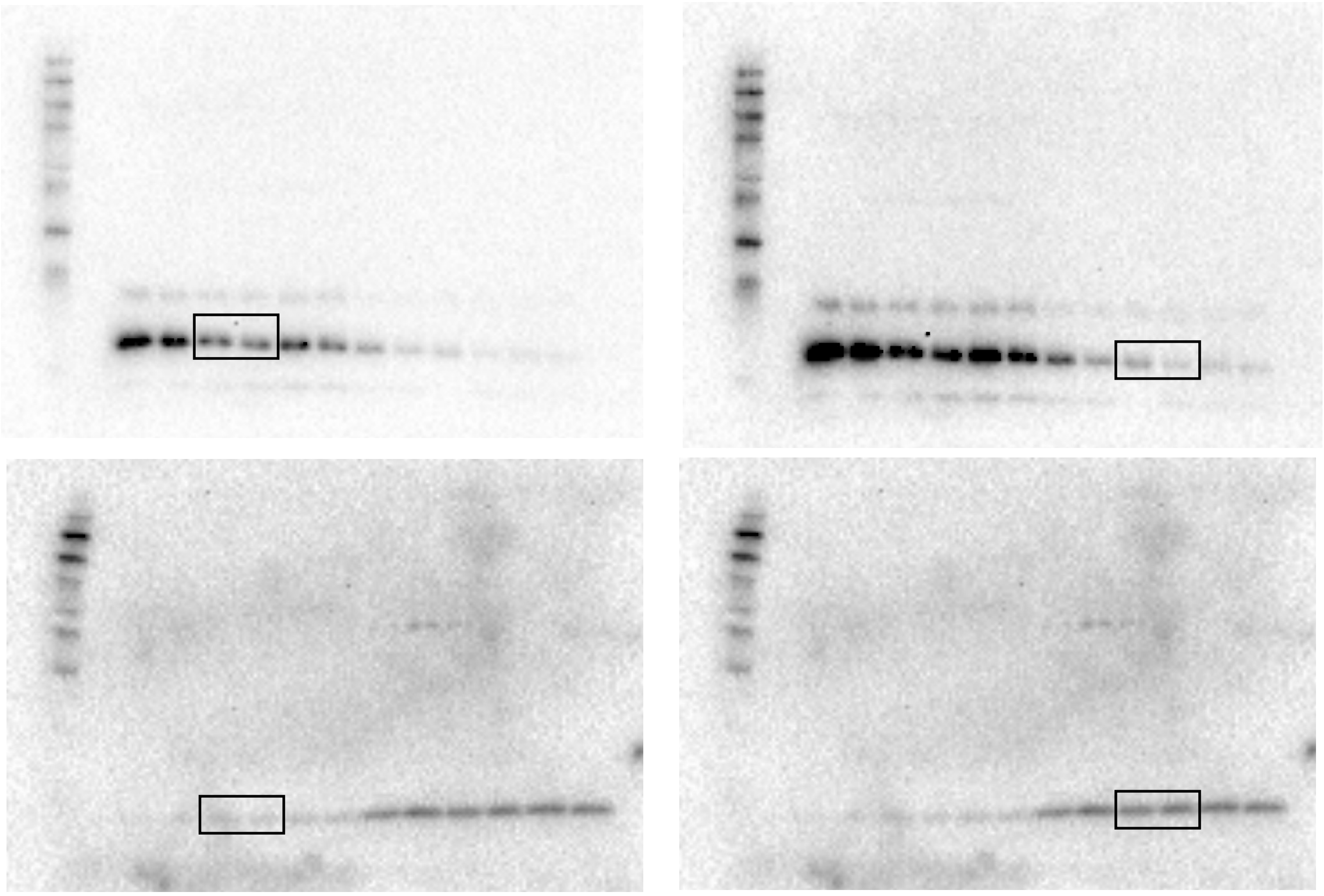

Supplementary Figure S3: Full length western blots used for Figure 4a. Caco-2 Cytosol (top left), Caco-2 Membrane (top right), Caco-2 Nucleus (bottom left) and Caco-2 Cytoskeleton (bottom right) western blots.

Claudin 2 Knock-in Protein Expression

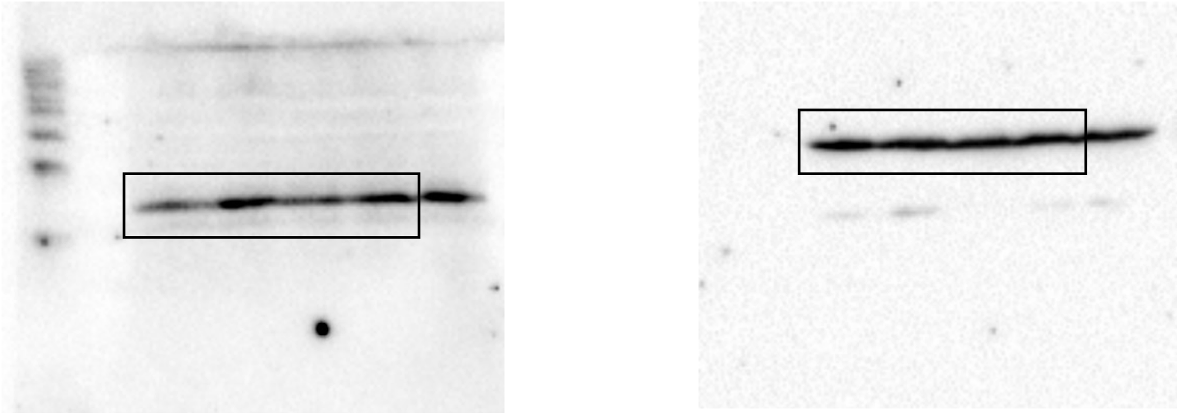

Supplementary Figure S4: Full length western blots used for Figure 5b. Claudin 2 (left) and β-actin (right) western blots.

Immunoprecipitation

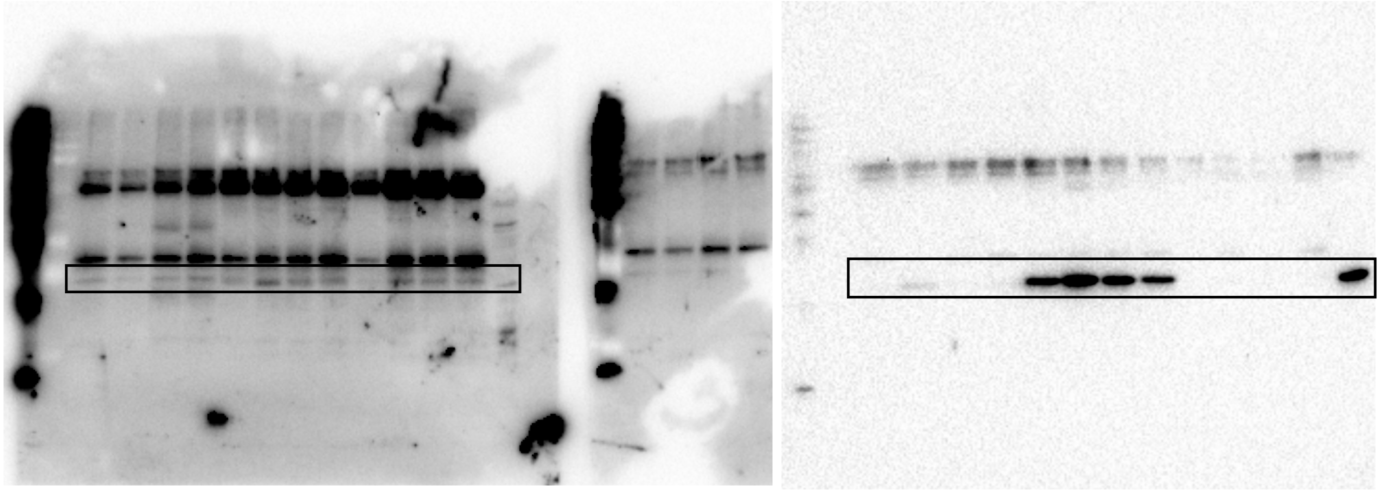

Supplementary Figure S5: Full length western blots used for Figure 7 a & b. Claudin 2 (left) and Caveolin 1 (right) western blots.
